# Three Distinct Subsets of Thymic Epithelial Cells in Rats and Mice Defined by Novel Antibodies

**DOI:** 10.1371/journal.pone.0109995

**Published:** 2014-10-21

**Authors:** Yasushi Sawanobori, Hiashi Ueta, Christine D. Dijkstra, Chae Gyu Park, Motoyasu Satou, Yusuke Kitazawa, Kenjiro Matsuno

**Affiliations:** 1 Department of Anatomy (Macro), Dokkyo Medical University, Tochigi, Japan; 2 Molecular Cell Biology and Immunology, VU University Medical Center Amsterdam, Amsterdam, Netherlands; 3 Laboratory of Immunology, Severance Biomedical Science Institute, Brain Korea 21 PLUS Project for Medical Science, Yonsei University College of Medicine, Seoul, Republic of Korea; 4 Department of Biochemistry, Dokkyo Medical University, Tochigi, Japan; University of Tokyo, Japan

## Abstract

**Aim:**

Thymic epithelial cells (TECs) are thought to play an essential role in T cell development and have been detected mainly in mice using lectin binding and antibodies to keratins. Our aim in the present study was to create a precise map of rat TECs using antibodies to putative markers and novel monoclonal antibodies (i.e., ED 18/19/21 and anti-CD205 antibodies) and compare it with a map from mouse counterparts and that of rat thymic dendritic cells.

**Results:**

Rat TECs were subdivided on the basis of phenotype into three subsets; ED18^+^ED19^+/−^keratin 5 (K5)^+^K8^+^CD205^+^ class II MHC (MHCII)^+^ cortical TECs (cTECs), ED18^+^ED21^−^K5^−^K8^+^ *Ulex europaeus* lectin 1 (UEA-1)^+^CD205^−^ medullary TECs (mTEC1s), and ED18^+^ED21^+^K5^+^K8^dull^UEA-1^−^CD205^−^ medullary TECs (mTEC2s). Thymic nurse cells were defined in cytosmears as an ED18^+^ED19^+/−^K5^+^K8^+^ subset of cTECs. mTEC1s preferentially expressed MHCII, claudin-3, claudin-4, and autoimmune regulator (AIRE). Use of ED18 and ED21 antibodies revealed three subsets of TECs in mice as well. We also detected two distinct TEC-free areas in the subcapsular cortex and in the medulla. Rat dendritic cells in the cortex were MHCII^+^CD103^+^ but negative for TEC markers, including CD205. Those in the medulla were MHCII^+^CD103^+^ and CD205^+^ cells were found only in the TEC-free area.

**Conclusion:**

Both rats and mice have three TEC subsets with similar phenotypes that can be identified using known markers and new monoclonal antibodies. These findings will facilitate further analysis of TEC subsets and DCs and help to define their roles in thymic selection and in pathological states such as autoimmune disorders.

## Introduction

The thymus, a lymphoid organ with a lobular structure, is important for the development of T cells. Specifically, thymocytes (T cell precursors) are subjected to both negative and positive selection in the thymus. Each lobule of the thymus has a cortex that contains densely packed CD4 and CD8 double-positive thymocytes and a medulla that contains sparser CD4 or CD8 single-positive thymocytes. Mainly in the cortex, thymocytes are subjected to positive selection, in which precursors with low reactivity to the MHC complex are deleted/eliminated. Subsequently, the thymocytes are subjected to negative selection in the medulla, a process that deletes/eliminates cells that have reactivity against self antigens [1].

Thymic epithelial cells (TECs) and thymic dendritic cells (tDCs) are considered to be responsible for the positive and negative selection of thymocytes. In mice and humans, cortical and medullary TECs (cTECs and mTECs) can be distinguished by means of expression of certain keratins and specific cell-surface molecules, or selective binding of *Ulex europaeus* lectin 1 (UEA-1). For example, CD205 [2]–[3], and Ly51 [4]–[6] are used to identify cTECs, and UEA-1 [7]–[8] and keratin 5 (K5) [7], [9]–[10] are recognized as mTEC markers. Keratin 8 (K8) [7], [9]–[10] is expressed in both the cortex and the medulla. Numerous studies have used these markers to describe the development or function of the thymus in mice, but few such studies have been conducted in other animals or in humans. Thus, the distribution and specificity of these markers in species other than mice remain largely unknown.

In addition to cTECs and mTECs, multinuclear cell structures called thymic nurse cells (TNCs) are found in isolated cell suspensions derived from the thymus [11]–[16]. For many years, it was unclear whether TNCs were a type of TEC that holds numerous thymocytes, or if they were structures that were somehow created during the cell-isolation procedure. Recently, TNCs were observed in vivo, and their role in T cell development was reported in mice [17]. However, TNCs in other species have not been widely studied.

The thymus also contains many dendritic cells (DCs) [1]. The role of thymic DCs (tDCs) in T cell development is still unclear, but studies on presentation of mTEC-derived antigens [18] have shown that tDCs are essential for the generation of naturally occurring regulatory T cells [19], although there is still room for debate. tDC subpopulations and the distribution of tDCs have not been reported in animals other than mice. Moreover, although the C-type lectin CD205 has been exploited as a marker of DC subsets [20], it is also expressed on cTECs [21]. Accordingly, mapping of tDCs remains incomplete.

The aim of this study was to create a precise map of rat TECs and compare it with that of mice using multicolor immunostaining. To characterize rat TECs, we used the newly generated monoclonal antibodies ED18, ED19, and ED21 and HD83 (raised against rat CD205), as well as antibodies that were reported previously to be reactive to rat antigens. Our results show that there are three TEC subsets in both rats and mice that have somewhat similar phenotypes in terms of reactivity with known and new antibodies. We also identified two distinct TEC-free areas that are unique to the rat thymus, and we discuss their possible roles in thymocyte development.

## Materials and Methods

### Animals

Inbred Lewis (RT1^l^), DA (RT1^a^) and PVG/c RT7^b^ (RT1^c^) rats (8–12 wks old) of both sexes and 8-wk-old male C57BL/6 mice were purchased from SLC Company. (Shizuoka, Japan). Animal handling and care protocols were approved by the Dokkyo Medical University Animal Experiments Committee, and were in accordance with Dokkyo University's Regulations for Animal Experiments and with Japanese Governmental Law No. 105. To obtain the thymus, all animals were killed by exsanguination from the abdominal aorta under isoflurane general anesthesia. At least three rats/mice were used in each experiment.

### Antibodies and reagents

The primary and secondary antibodies used for immunohistology and flow cytometric analysis are listed in Tables 1 and 2 respectively. ED monoclonal antibodies were prepared by immunizing mice with rat splenic stromal components, and the hybridomas were produced using standard methods [22]. Clones reactive to thymus but not to spleen or lymph nodes were screened and established as the ED18, 19, and 21 clones. The anti-CD205 monoclonal antibody HD83 was produced at Rockefeller University in New York [20].

**Table 1:** Primary antibodies used in this research.

Antigen	Isotype	Clone	Conjugate	Source
unknown	mouse IgM	ED18	−/biotin#/Alexa488^#/^Alexa594#	a
unknown	mouse IgM	ED19	−/biotin#/Alexa488^#/^Alexa594#	a
unknown	mouse IgM	ED21	−/biotin#/Alexa488#/Alexa594#	a
keratin 5	rabbit IgG	polyclonal	−	b
keratin 8	chicken IgY	polyclonal	−	c
rat CD45	mouse IgG_1_	OX1	−/PE	d/e
rat CD103	mouse IgG_1_	OX62	−	f
rat CD205	mouse IgG_1_	HD83	−	g
rat MHCII (RT1B^l^)	mouse IgG_1_	OX3	−/Alexa647#	h
mouse CD205	rat IgG_2a_	NLDC145	−	i
mouse MHCII	rat IgG_2b_	M5/114	biotin#/Alexa647#	j
autoimmune regulator (AIRE)	goat IgG	polyclonal	−	k
claudin-3	rabbit IgG	polyclonal	−	l
claudin-4	rabbit IgG	polyclonal	−	l
*Ulex europaeus* Lectin 1 (UEA-1)	−	lectin	biotin/FITC	m
isotype control	mouse IgG	polyclonal	−	n

# : conjugated with either biotin conjugation kit (Dojindo) or Alexa conjugation kit (Invitrogen) in house, ^a^ produced at VU University Medical Center (Amsterdam, Netherland), ^b^ Assay Biotechnology (Sunnyvale, CA, USA), ^c^ Abcam (Cambridge, United Kingdom), ^d^ BD Pharmingen (Franklin Lakes, NJ, USA), ^e^ Biolegend (San Diego, CA, USA), ^f^ donated by Dr. M. Brenan, ^g^ produced at Rockefeller University (NY, USA), ^h^ ECACC, ^i^ donated by Dr. G. Kraal, ^j^ eBioscience (San Diego, CA, USA), ^k^ Sigma-Aldrich (Saint Louis, MO, USA), ^l^ Invitrogen (Camarillo, CA, USA), ^m^ Vector Labs (Burlingame, California, CA, USA), ^n^ Jackson Immunoresearch (West Grove, Pennsylvania, USA)

**Table 2:** Secondary/tertiary antibodies used in this research.

product	conjugate	source
goat anti-mouse IgM	horse radish peroxidase	MP biomedicals (Santa Ana, California, USA)
horse anti-mouse IgG	biotin	Vactor Labs
goat anti-chicken IgY	alkaline phosphatase	Jackson Immunoresearch
donkey anti-chicken IgY	Alexa594	Jackson Immunoresearch
donkey anti-goat IgG	Alexa594	Jackson Immunoresearch
donkey anti-rabbit IgG	alkaline phosphatase	Jackson Immunoresearch
goat anti-rabbit IgG	horse radish peroxidase	MP biomedicals
donkey anti-rabbit IgG	aminomethyl coumarin acetate (AMCA)	Jackson Immunoresearch
donkey anti-rat IgG	alkaline phosphatase	Jackson Immunoresearch
donkey anti-biotin	alkaline phosphatase	Sigma-Aldrich
goat anti-biotin	Alexa488#	Sigma-Aldrich
streptavidin	Alexa488, 594	Invitrogen
streptavidin	PerCP-Cyanin5.5	eBioscience

# : conjugated with Alexa conjugation kit (Invitrogen) in house.

### Cell isolation

TNCs were isolated as reported previously [15], Briefly, thymic tissues were digested with 0.28% collagenase D (Roche Diagnostics, Indianapolis, IN, USA) in HBSS supplemented with 0.01% DNase I (Roche) in a 37°C water bath with gentle shaking for 25 min. For the final 5 min of incubation, 1 mM EDTA (Wako Pure Chemical Industries, Ltd., Osaka, Japan) was added. The digested cell suspension was centrifuged and resuspended in PBS(−), then overlaid onto heat-inactivated fetal calf serum. After 10 min at room temperature, the serum layer was recovered, centrifuged, and once more resuspended in PBS(−). This sedimentation procedure was repeated four times. Several thousand TNCs were obtained from each rat thymus and used for immunocytochemistry.

For flow cytometric analysis of TECs, thymi were minced with scissors and incubated with gentle rotation in RPMI (Life Technologies, Carlsbad, CA, USA) supplemented with 10% fetal calf serum at 4°C for 30 min to remove loosely-bound thymocytes. Debris was collected on 40 µm filters (BD Falcon, San Jose, CA, USA) and digested with 0.0125% Liberase TH (Roche) in HBSS supplemented with 0.02% DNase I at 37°C in a water bath with gentle shaking for 35 min. Each digestion reaction was pipetted with a Pasteur pipette every 10 min. For the final 5 min of incubation, 1 mM EDTA was added. The digested cell suspension was centrifuged and resuspended in 15% OptiPrep (Axis-Shield, Oslo, Norway)/PBS(−) in centrifuge tubes, and 11% OptiPrep/PBS(−) and PBS(−) were overlaid on the cell suspension. The tubes were centrifuged at 600×g for 25 min at 25°C. The large cells at the interface between the 11% OptiPrep/PBS(−) and PBS(−) were collected and subjected to flow cytometric analysis.

### Immunohistochemistry and immunocytochemistry

Sections of freshly frozen tissues and cytosmears were fixed in acetone and immunostained as described previously [23]–[24]. For light microscopy, molecules of interest were stained blue using alkaline phosphatase-conjugated secondary/tertiary antibodies and Vector Blue substrate (Vector Laboratories, Burlingame, CA, USA). Additionally, type IV collagen, which reveals the framework of tissues was stained brown with peroxidase-conjugated secondary antibody and 3, 3′-diaminobenzidine substrate (Dojindo Molecular Technologies, Kumamoto, Japan). Photomicrographs were captured with a Microphot-FX microscope (Nikon, Tokyo, Japan) and a DP26 digital camera (Olympus, Tokyo, Japan). The proportions of the cortical area and two medullary subareas (described in Results) were calculated in the rat thymus with digital image analysis software (cellSens, Olympus). Thymi from three male Lewis rats were used for this purpose.

For fluorescence microscopy, fluorescent dye-conjugated secondary antibodies or streptavidin were used. Multichannel color fluorescence images were captured with an Axioskop 2 Plus fluorescence microscope equipped with an AxioCam MRm or MRc5 camera (Zeiss, Oberkochen, Germany). We assigned pseudocolors to each channel to make merged images more comprehensible by maximizing contrast using AxioVision software (Zeiss).

### Flow cytometric analysis

Cells were stained using conventional methods. To stain intracellular antigens, cells were permeabilized with IC Fixation Buffer and Permeabilization Buffer (eBioscience, San Diego, CA, USA), following the manufacturer's instruction. Stained cells were analyzed with a FACSCalibur flow cytometer (Becton Dickinson, Franklin Lakes, NJ, USA) and Flowjo software (TreeStar Inc., Ashland, OR, USA).

### Western blot analysis of ED monoclonal antibodies

Frozen rat thymus (380 mg) was homogenized by using Polytron homogenizer (Kinematica AG, Luceme, Switzerland). After centrifugation at 10,000×g for 30 min, 20 µg of the lysate was separated by SDS-PAGE. Membranes were incubated with the primary antibodies (ED18, ED19, and ED21) for 2 hours at room temperature. Subsequently, the membranes were washed and incubated with anti-IgM conjugated with peroxidase for 1 h at room temperature, then the immunopositive proteins were detected by addition of the West Pico blotting reagent and exposure to X-ray film (Thermo Fisher Scientific, Waltham, MA, USA). This procedure was repeated twice.

## Results

### Immunohistological profiles of TECs

**Figure 1:**
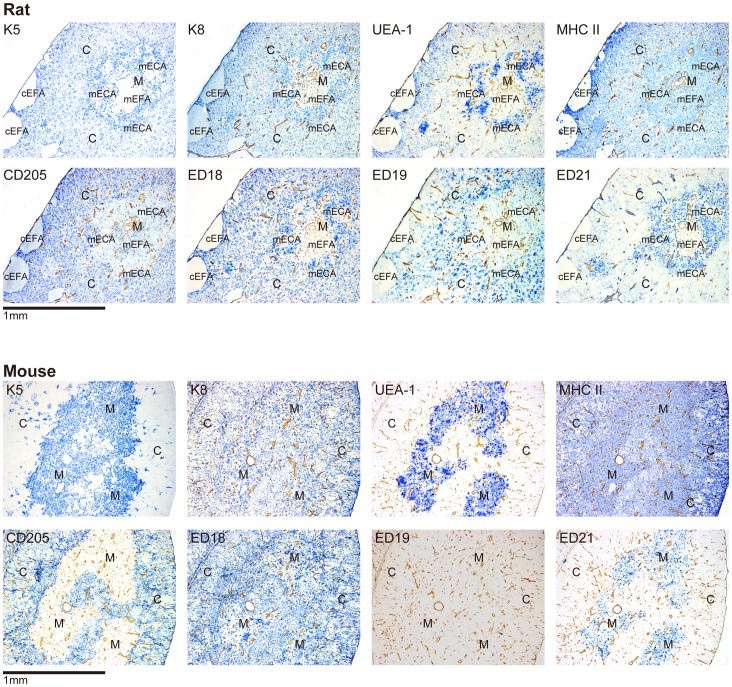
Immunohistological analysis of thymic epithelial cell-associated molecules in the thymi of rats and mice. Double immunoenzyme staining for molecules of interests (blue) and tissue frameworks (type IV collagen, brown) Cortical (subcapsular) and medullary epithelium-free areas unique to rats are indicated by “cEFA” and “mEFA”, respectively. Epithelium-containing areas in the medulla are indicated by “mECA”. The antibodies used were anti-K5 followed by anti-rabbit IgG, anti-K8 followed by anti-chicken IgY, and anti-mouse CD205 followed by anti-rat IgG. Anti-rat MHCII and anti-rat CD205 followed by biotin-conjugated anti-mouse IgG, and biotin-conjugated anti-mouse MHCII, and biotin-conjugated ED18/ED19/ED21 were further reacted with anti-biotin antibody. Secondary antibodies and anti-biotin antibody were all conjugated with alkaline phosphatase and developed with the Vector Blue substrate kit. Tissue frameworks were stained with anti-type IV collagen antibody followed by peroxidase-conjugated anti-rabbit IgG and developed with 3, 3′-diaminobenzidine substrate.

We first tested the reactivity of rat and mouse thymic sections with the newly developed ED18, ED19, ED21, and HD83 antibodies and a panel of antibodies specific to a selection of putative TEC antigens (Fig. 1). In mice, anti-K5 antibody stained mainly the medulla [7], [9]–[10], while in rats, anti-K5 and anti-K8 antibodies stained both the cortex and the medulla. UEA-1, a lectin that specifically binds to mTECs in mice [7]–[8], selectively stained the mouse medulla but strongly stained the medulla and weakly stained the cortex of rats. An anti-MHC class II (MHCII) antibody showed a reticular staining pattern in the cortex and strong staining of round or polygonal cells in the medulla of both rats and mice. As reported previously, CD205 is expressed not only by cTECs but also by the DC subset in rats and humans as well as in mice [20]–[21]. In the present study, anti-rat CD205 (HD83) and anti-mouse CD205 (NLDC 145) antibodies stained the cortex of both species in a pattern similar to that of the anti-K8 and anti-MHCII antibodies and also stained DC-like cells in the medulla. ED18 stained both the cortex and the medulla of rats and mouse. ED19 staining was restricted to the rat cortex, and there was no staining in the mouse thymus. ED21 stained both the rat and mouse medulla, and weakly stained the rat cortex. Anti-K5, anti-K8, UEA-1, anti-MHCII, anti-CD205, ED18, ED19, and ED21 antibodies all had reticular staining patterns in the cortex. In the medulla, anti-K5, anti-K8, UEA-1, ED18, and ED21 antibodies stained fusiform cells. These results are summarized in Table 3. We observed subcapsular and medullary epithelium-free areas (cEFAs and mEFAs, respectively in Fig. 1). cEFAs were reported on previously [25]–[26]. mEFAs and medullary TEC-containing areas (mECAs) are described in detail below.

**Table 3:** Expression of TEC-related markers in thymi of rats and mice.

	Rat		Mouse	
	cortex	medulla	cortex	medulla
Keratin5	+	+	few	+
Keratin8	+	+	+	+
UEA-1	dull	+	−	+
MHCII	+	+	+	+
CD205	+	+	+	+
ED18	+	+	+	+
ED19	+	−	−	−
ED21	dull	+	−	+

### Characterization of cTECs

**Figure 2:**
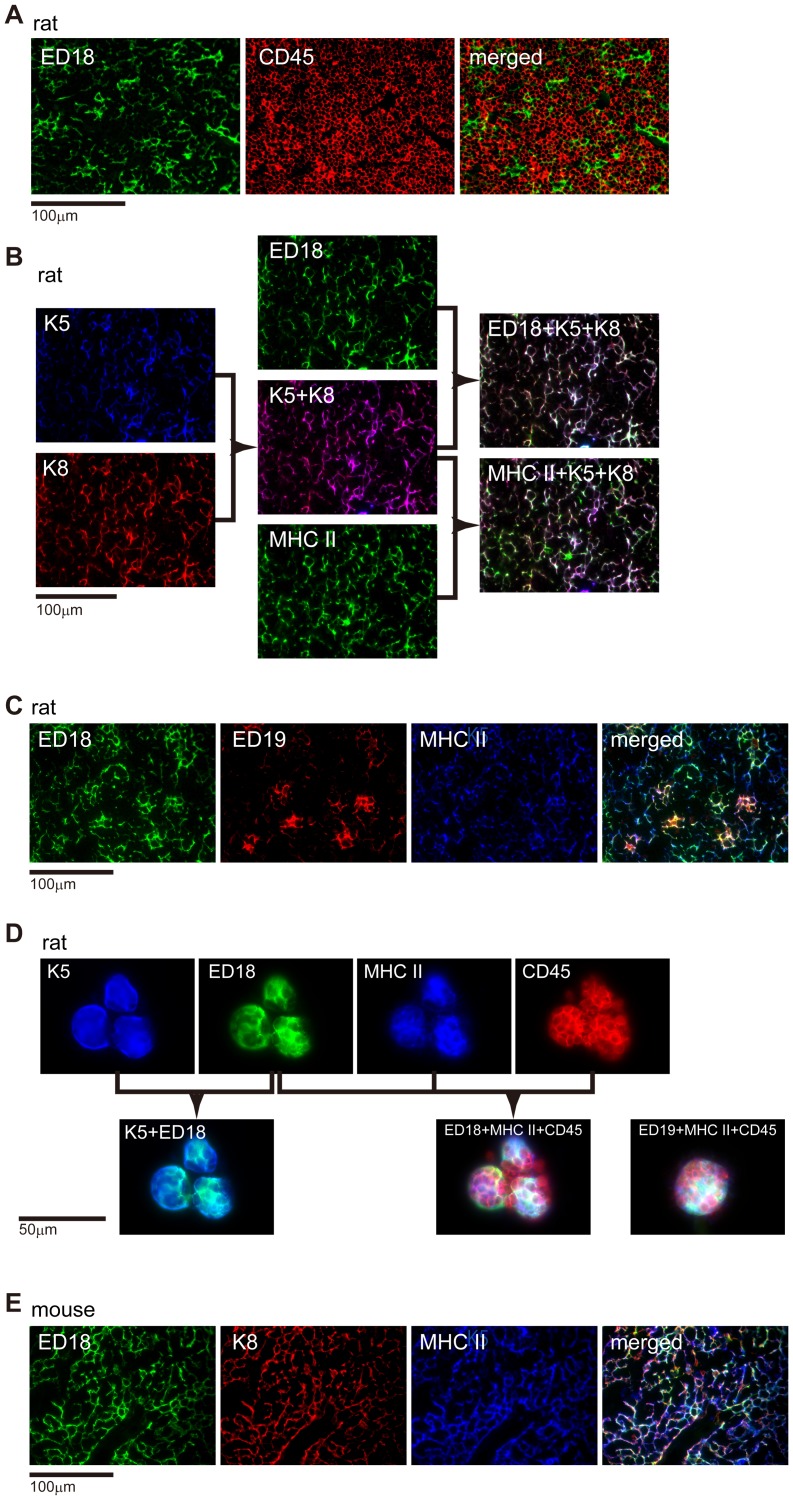
Characterization of cortical thymic epithelial cells. Frozen sections of rat (A-C), and mouse (E) thymus, and a cytosmear from isolated rat thymic nurse cells (D) were subjected to multicolor immunofluorescence staining. The following antibodies were used: Alexa488-conjugated ED18, Alexa488-conjugated ED19, anti-rat CD45 antibody followed by Alexa594-conjugated anti-mouse IgG antibody, anti-K5 antibody followed by AMCA-conjugated anti-rabbit IgG antibody, anti-K8 antibody followed by Alexa-594 conjugated anti-chicken IgY antibody, Alexa647-conjugated anti-rat MHCII antibody and Alexa647-conjugated anti-mouse MHCII antibody.

We used multicolor fluorescent immunohistochemistry to confirm that ED18^+^ and ED19^+^ cells in the rat thymic cortex were cTECs (Fig. 2). Both ED18^+^ and ED19^+^ cells were negative for CD45 (leukocyte common antigen), showing that they were not thymocytes derived from the bone marrow (ED18 shown in Fig. 2A, ED19 not shown). ED18 staining was almost identical to that of anti-MHCII, anti-K5, and anti-K8 antibodies (Fig. 2B). When ED18 and ED19 were used to stain the same section, ED19 stained relatively fewer cells than did ED18. ED19^+^ cells were large and had branched cytoprocesses that included many thymocytes, suggesting that these cells may correspond to TNCs [15]–[16] (Fig. 2C). CD205 was mostly coexpressed with the ED18 epitope in the cortex (Fig. S1), but not in the medulla. These results indicate that rat cTECs are ED18^+^ ED19^+/−^K5^+^K8^+^CD205^+^MHCII^+^. Staining patterns for UEA-1 and ED21 in the rat cortex (Fig. 1) were so weak that they were cut off in fluorescent images; therefore we could not investigate their expression on cTECs in detail. These results are summarized in Table 4.

**Table 4:** Phenotype of TEC subsets in rats and mice.

	Rat			Mouse		
	cTEC	mTEC1	mTEC2	cTEC	mTEC1	mTEC2
Keratin5	+	−	+	few	−	+
Keratin8	+	+	dull	+	+	dull
UEA-1	dull?	+	−	−	+	−
MHCII	+	many	few	+	many	few
CD205	+	−	−	+	NT#	NT
ED18	+	+	+	+	+	+
ED19	+/−	−	−	−	−	−
ED21	dull?	−	+	−	−	+
Claudin3/4	−	many	few	−	many	few
AIRE	−	many	few	−	many	few

# : not tested.

We next compared the phenotypes of isolated TNCs and cTECs. Multicolor staining revealed ED18^+^ED19^+/−^K5^+^MHCII^+^ cells that formed a shell-like structure and contained CD45^+^ thymocytes within (Fig. 2D). Staining with other combinations of antibodies revealed that K8 and CD205 were also expressed by the shell-like cells (data not shown). These results suggest that TNCs are structures that are derived from cTECs. In mice, ED18 also showed a staining pattern identical to that of anti-K8 and anti-MHCII (Fig. 2E).

As noted above, CD205 was expressed by cTECs in the cortex, but not by mTECs (Fig. 1 and Fig. S1A). Because CD205 is also a marker for DC subsets, cortical DCs may be ED18^−^CD205^+^MHCII^+^ cells. However, only ED18^−^CD205^−^MHCII^+^ cells, but not ED18^−^CD205^+^MHCII^+^ cells, were detected in the cortex, and CD103 was expressed on MHCII^+^ cells (Fig. S1B). This indicates that DCs in the cortex are MHCII^+^CD103^+^CD205^−^.

### Characterization of mTECs

**Figure 3:**
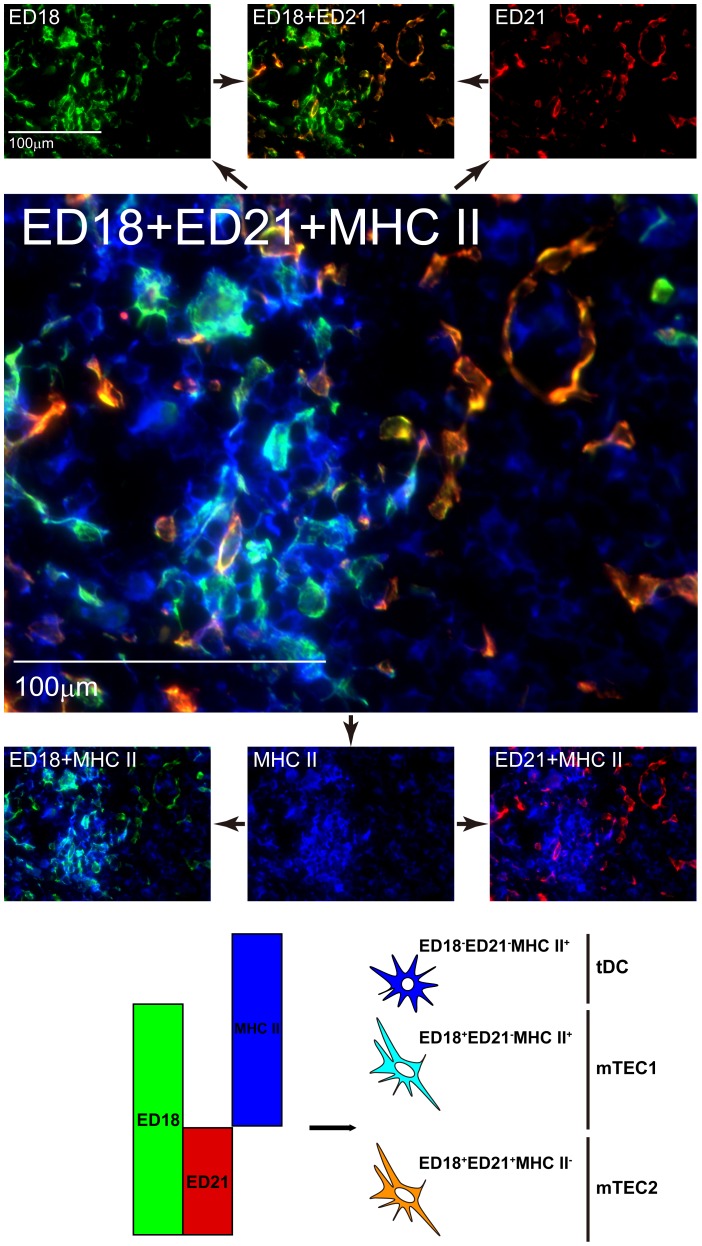
Characterization of rat medullary thymic epithelial cells 1: relationship between ED18 and ED21 staining. A section of thymus from a Lewis rat was stained with Alexa594-conjugated ED18, Alexa488-conjugated ED21, and Alexa647-conjugated anti-rat MHCII antibody. Pseudocolors were adjusted and assigned with AxioVision software. Merged images are explained schematically below the panels.

**Figure 4:**
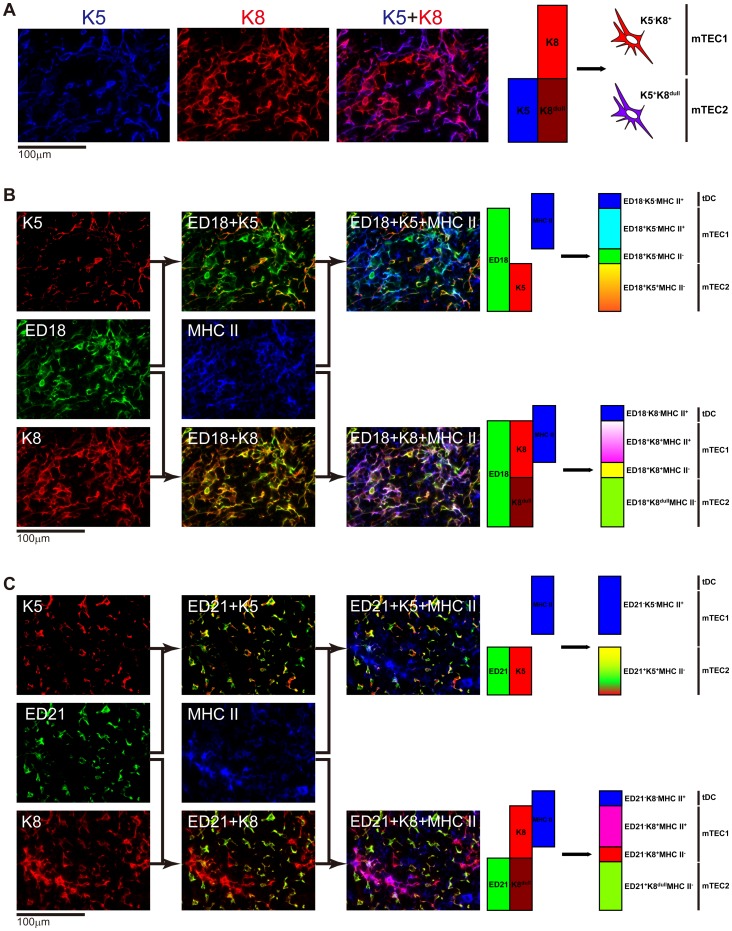
Characterization of rat medullary thymic epithelial cells 2: relationship between keratin expression and ED18/ED21. Sections of thymus from a Lewis rat were stained with Alexa488-conjugated ED18 (A, B) or Alexa488-conjugated ED21 (C). Subsequently, the sections were stained with Alexa647-conjugated anti-rat MHCII antibody, anti-K5 and anti-K8 followed by AMCA-conjugated anti-rabbit IgG and Alexa594-conjugated anti-chicken IgY antibodies, respectively. Pseudocolors were adjusted and assigned with AxioVision software. Pictures in (A) and (B) are from the same multicolor picture with different color assignments. Merged images are explained schematically below the panels.

To characterize mTECs further, we strained rat medullary stromal cells with several combinations of antibodies. When rat ED18, ED21, and anti-MHCII antibodies were applied (Fig. 3), mTECs could be divided into two subsets: ED18^+^ED21^−^ and ED18^+^ED21^+^. We called these two subsets mTEC1 and mTEC2, respectively. MHCII was expressed mainly on the mTEC1 subset. We next investigated expression of keratins on mTEC1 and mTEC2 (Fig. 4). K8 was expressed with two different intensities: K8^+^ and K8^dull^, and K5^+^ cells were almost identical to K8^dull^ cells (Fig. 4A). When ED18, anti-K5, anti-K8, and anti-MHCII antibodies were applied to the same sections, ED18^+^ cells in the medulla included both K5^−^K8^+^ and K5^+^K8^dull^ cells, and MHCII was preferentially expressed on the K5^−^K8^+^ subset (Fig. 4B). On the other hand, ED21^+^ cells were almost identical to the K5^+^K8^dull^ subset and rarely expressed MHCII (Fig. 4C).

**Figure 5:**
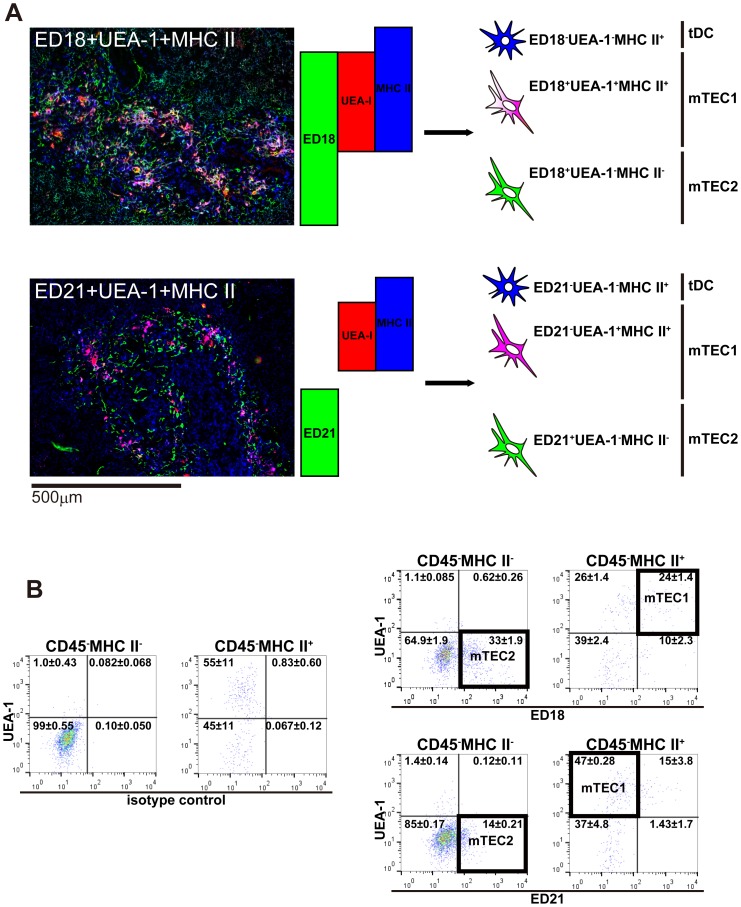
Characterization of rat medullary thymic epithelial cells 3: UEA-1 binding to mTEC subpopulations. (A) A section of a thymus from a Lewis rat was stained with Alexa594-conjugated ED18 or ED21, Alexa647-conjugated anti-rat MHCII antibody, and biotin-conjugated UEA-1 followed by Alexa488-conjugated anti-biotin antibody. Pseudocolors were assigned using AxioVision software. (B) The low-density fraction from Lewis rat thymi was subjected to flow cytometric analysis. Cells were stained with Alexa488-conjugated control IgM or ED18/ED21, anti-rat CD45-PE, biotin-conjugated UEA-1 followed by streptavidin-PerCP-Cy5.5, and Alexa647-conjugated anti-rat MHCII.

Because binding to UEA-1 has been used as a marker for mTECs [7]–[8], we examined relationships between UEA-1 and ED18/ED21. When combinations of ED18, UEA-1, and anti-MHCII, or ED21, UEA-1, and anti-MHCII were used to stain the same section, UEA-1 stained a subset of ED18^+^ cells, and MHCII was expressed on these ED18^+^UEA-1^+^ cells (Fig. 5A). On the other hand, UEA-1^+^MHCII^+^ cells were ED21^−^.

Above all, these data indicate that rat mTECs can be divided into two subsets: ED18^+^ED21^−^K5^−^K8^+^UEA-1^+^ mTEC1s and ED18^+^ED21^+^K5^+^K8^dull^UEA-1^−^ mTEC2s. mTEC1s and mTEC2s were detected by flow cytometric analysis as MHCII^+^UEA-1^+^ED18^+^ED21^−^ cells and MHCII^−^UEA-1^−^ED18^+^ED21^+^ cells, respectively (Fig. 5B).

When mouse thymi were compared with rat data (except for flow cytometric analysis), these populations were phenotypically roughly similar in rats (Figs. 3–5) and mice (Figs. S2–S4; Table 4).

**Figure 6:**
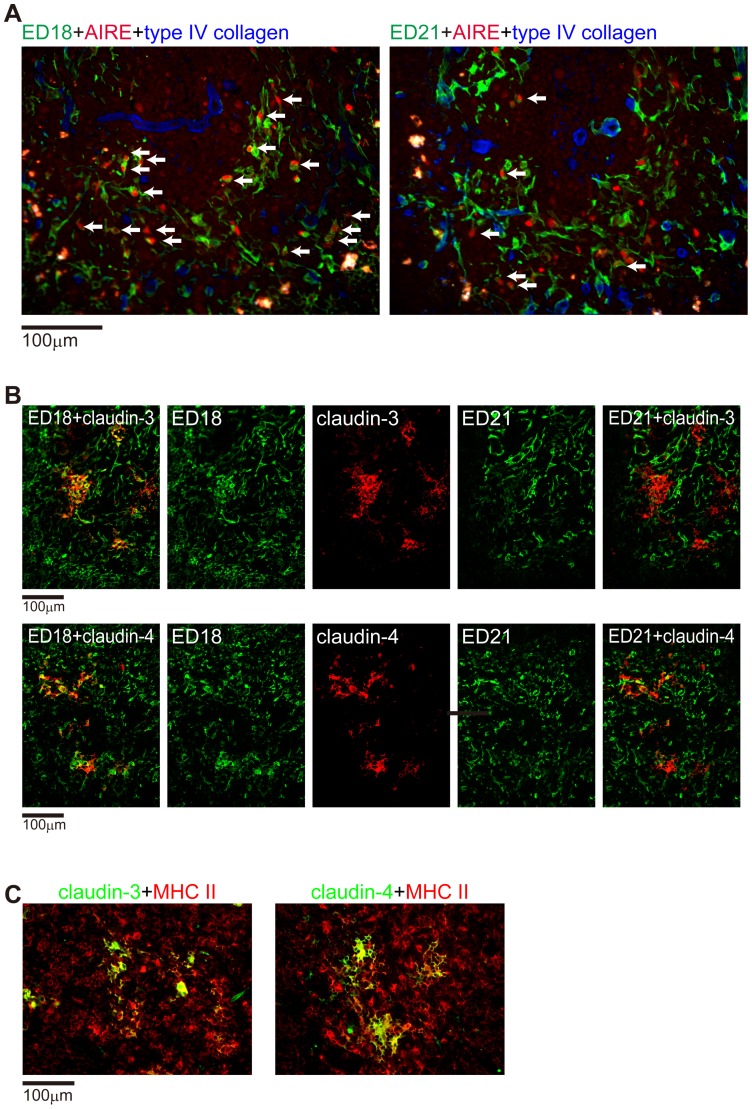
Expression of functional molecules in rat mTEC1 and mTEC2 subsets. (A) Rat thymic sections were stained with Alexa488-conjugated ED18 or ED21 (green) and anti-AIRE antibody followed by Alexa594-conjugated anti-goat IgG (red). Tissue framework was stained blue with anti-type IV collagen followed by AMCA-conjugated anti-rabbit IgG. Arrows indicate AIRE expression associated with ED18- and ED21-positive cells. (B, C) Rat thymic sections were stained with anti-claudin-3 or anti-claudin-4 antibodies followed by AMCA-conjugated anti-rabbit IgG, Alexa488-conjugated ED18, Alexa594-conjugated ED21, and Alexa647-conjugated anti-rat MHCII. Pseudocolors were assigned using AxioVision software.

Next, we tried to identify some of the functional molecules in the cells of each of the mTEC subsets (Fig. 6). Autoimmune regulator (AIRE), a transcriptional factor required for negative selection [27], was expressed preferentially in nuclei of ED18^+^ cells (Fig. 6A). Claudin-3 and claudin-4, tight junction components that are expressed by AIRE^+^ TECs and their precursors [28], were expressed preferentially on ED18^+^ cells (Fig. 6B). Claudin-3^+^ or claudin-4^+^ TECs were also MHCII^+^ (Fig. 6C). Taken together, these data suggest that mTEC1 cells are an “active” subset compared to mTEC2 cells. The preferential expression of AIRE on the mTEC1 subset was also confirmed on mouse thymi (Fig. S5). These findings are shown in Table 4.

### Epithelium-free areas and DCs in the rat thymic medulla

**Figure 7:**
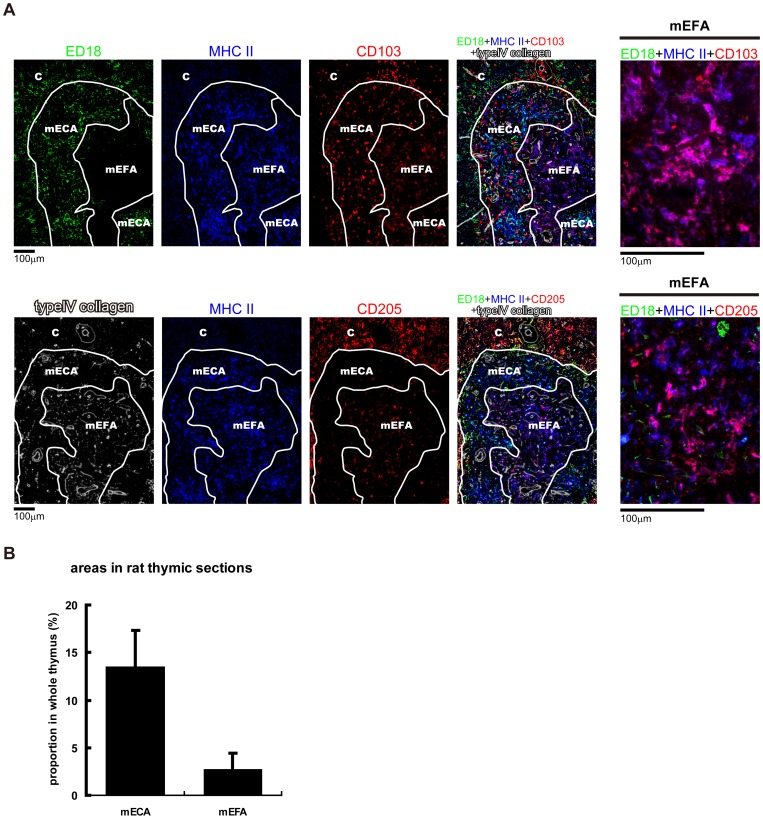
Epithelium-free areas in the rat thymic medullas. (A) Frozen sections of Lewis rat thymi were stained with anti-CD205 or anti-CD103 antibodies followed by biotin-conjugated anti-mouse IgG antibody and Alexa488-conjugated anti-biotin antibody, Alexa-594 conjugated ED18 antibody, Alexa-647 conjugated anti-rat MHCII antibody, and anti-type IV collagen antibody followed by AMCA-conjugated anti-rabbit IgG antibody. Pseudocolors were assigned using AxioVision software. (B) Whole images of ED21- stained thymic sections from three Lewis rats were captured, and mECAs and mEFAs were calculated with cellSense software.

As shown in Figure 1, the rat thymic medulla had an area that completely lacked mTEC marker-positive cells. Other parts of the medulla were packed with mTECs (Fig. 1, 7A). We called these areas mEFAs, mECAs, respectively. Compared to the mECAs, the mEFAs had delicate networks of type IV collagen fibers. MHCII^+^ cells were diffusely present in both areas. The TEC-free areas were found in various common rat strains, including Lewis, DA, and PVG/c rats, but were not found in mice (data not shown). Proportions of the cortical area, mECAs, and mEFAs in the whole thymus were approximately 83%, 14%, and 3%, respectively (Fig. 7B). This indicates that the mEFAs occupy approximately one-fifth of the medulla.

When we investigated the presence of DCs in the medulla, we observed CD103 expression in both the mECAs and mEFAs, whereas CD205^+^cells accumulated in the mEFAs (Fig. 7A). Expression of CD103 and CD205 exactly overlapped with that of MHCII, suggesting that they are expressed on thymic DCs. These epitopes were expressed by both MHCII^+^ and MHCII^−^ cells in the mECAs, but mainly by MHCII^+^ cells in the mEFAs.

### Western blot analysis of ED monoclonal antibodies

By western-blot analysis of whole thymic lysate (Fig. S6), the immunoreactivities were successfully detected by each antibodies (ED18, ED19, and ED21). In case of ED19 or ED21, there seemed to be a single band corresponding to a molecular weight 46 kDa or 50 kDa, respectively, whereas several weaker signals were spreading between 75 to 25 kDa in case of ED18.

## Discussion

Our study revealed the following findings: (1) Rat TECs can be divided into three subsets: ED18^+^ED19^+/−^K5^+^K8^+^CD205^+^MHCII^+^ cTECs, ED18^+^ED21^−^K5^−^K8^+^UEA-1^+^CD205^−^ mTEC1s, and ED18^+^ED21^+^K5^+^K8^dull^UEA-1^−^CD205^−^ mTEC2s. (2) ED18 and ED21 antibodies were cross reactive with mouse proteins, and mTEC1 and mTEC2 were also found in mice. (3) TNCs were defined in cytosmears as a subset of cTECs that were ED18^+^ED19^+/−^K5^+^K8^+^. (4) mEFAs unique to rats were found. (5), Medullary DCs were MHCII^+^CD103^+^CD205^+^ in mEFAs and MHCII^+^CD103^+^CD205^−^ in mECAs.

Keratins are intermediate filament components, and thus they are intracellular proteins. ED18 and ED21 epitopes were detectable by flow cytometric analysis after membrane permeabilization (Fig. 5B) but could not be detected on intact cells (data not shown), suggesting that these epitopes are on intracellular proteins. Moreover, when cryosections were digested with collagenase, the antigenicity of both keratins and ED18, ED19, and ED21 epitopes disappeared (data not shown). In addition, western blot analysis demonstrated that molecular weight of ED19 and ED21 epitopes is around 50 kDa, which is within the range of keratin families (40–70 kDa). Taken together, these findings suggest that the epitopes for these antibodies are parts of keratin-related molecules.

In contrast to mTECs, cTECs were not detectable by flow cytometric analysis, even after permeabilization. However, immunostaining of cytosmears of isolated TNCs with ED18, ED19, ED21, anti-K5, and anti-K8 antibodies showed clearly stained TNCs (Fig. 2D). This may be because TNCs hold many thymocytes within a broad, shell-like cell membrane that protects the contents from digestion. ED19 stained a limited population of ED18^+^ cTECs in the rat cortex, that had a TNC-like structure, although isolated TNCs included not only ED19^+^ but also ED19^−^ populations. Thus, ED19 may be selectively expressed by the TNC subset, but new discrete markers are required to investigate this possibility.

We observed that MHCII was preferentially expressed in mTEC1s (Fig. 3 and Table 4), suggesting that these cells are the “active” subset. We also observed that AIRE, claudin-3, and claudin-4, which are tight junction components expressed on AIRE^+^ TECs and their precursors in mice [28], were expressed preferentially on rat mTEC1s (Fig. 6). It is possible that the keratin expression pattern is altered during the maturation of mTECs. Although K5 has long been exploited as an indicator of medullary areas in mice, some K5^+^K8^+^ TECs are also present in the cortex and are thought to be progenitors that develop into cortical and medullary cell subsets [29]. However, cTECs in rat thymi were solely K5^+^K8^+^; thus, other marker molecules are needed to explore this hypothesis in rats. On the other hand, K8 is also expressed in some K5^+^/K14^+^ mTECs and others have hypothesized that K5 and K14 expression is lost during terminal differentiation [30]. It is possible that the K5^+^K8^dull^ED21^+^ mTEC2 population that we identified in rats and mice (Table 4) corresponds to these progenitors. There may be mTECs that consist of different maturation stages (or active and resting subsets) that can be identified using a combination of these antibodies, and these may be present in both rats and mice. In particular, the, ED21 monoclonal antibody may be specific antibody to immature (or resting) mTECs because its staining is limited to the medulla. In humans, pan-keratin antibodies are used routinely to identify TECs, but only a few papers have reported the localization of each kind of keratin. One study showed that K14 was expressed in mTECs and subcapsular cTECs [31], and another showed that K5 was expressed throughout the cortex and medulla [32]. However, the relationships between function, maturation, and keratin expression patterns are still unclear in humans.

We found EFAs in the rat thymus, both in the cortex (Fig. 1) and in the medulla (Fig. 7). mEFAs are rarely mentioned in the literature. In the present study, mEFAs were commonly found in several rat strains and occupied approximately one-fifth of the medulla in Lewis rats, indicating that they are a distinct structure. Interestingly, CD205^+^MHCII^+^ DCs tended to accumulate in the mEFAs, whereas CD103^+^MHCII^+^ DCs were found throughout the mECAs and mEFAs (Figs. 7). Both a lack of tDCs [33]–[35] and AIRE deficiency [36] are reported to lead to autoimmunity. Others have reported that tDCs present antigens derived from mTECs [18]. Therefore, these cell types seem to cooperate for optimal induction of self tolerance, and some transport system for autoantigens from mTECs in the mECAs to tDCs in the mEFAs might exist. Based on some reports suggesting that tDCs are responsive in regulatory T cell generation [37]–[39], we assumed that regulatory T cells may be unevenly distributed in the mECAs or mEFAs. However, immunohistochemistry revealed that FoxP3^+^ cells were distributed evenly in both areas (data not shown). TCRαβ^+^, CD4^+^, and CD8^+^ cells were also randomly distributed in both areas. At least three subsets of tDCs have been described in mice [40]–[41], but CD205 expression was not investigated in these three subsets. Interestingly, a CD205-IgG fusion protein showed that apoptosis-induced thymocytes, but not intact thymocytes, express the CD205 ligand [42]. Accordingly, mEFA areas in the rat thymic medulla may be a compartment in which CD205^+^ DCs remove apoptosis-induced thymocytes as part of negative selection.

cEFAs were described previously as epithelium-free areas containing CD4^+^CD8^+^TCRαβ^+^ thymocytes and macrophages [25]–[26] and DCs (Sawanobori, unpublished data). Although it is hypothesized that the cEFAs are “ waiting rooms” for thymocytes during the selection process, their exact role remains unclear [25].

From a functional perspective, the mECAs, mEFAs, mTEC1s, and mTEC2s might be subjected to different influences in pathological states; such differences could be associated with the onset of disorders. For example, we found that the mEFAs and mTEC1s were decreased in graft-versus-host disease and after cyclosporine administration (unpublished data). Certain subsets of TECs and substructures might be implicated in disease pathology and thus may be potential therapeutic targets. Our new antibodies provide new tools with which to study TECs during development and aging, and during pathological conditions. Further studies are required to investigate the expression of keratin-like molecules during TEC maturation and differentiation.

## Supporting Information

Figure S1 **CD205 expressed on cortical thymic epithelial cells in the cortex and dendritic cells in the medulla.** (A) A rat thymus was stained with anti-CD205 followed by Alexa594-conjugated anti-mouse IgG and Alexa488-conjugated ED18. C, cortex; M, medulla; cEFA, cortical (subcapsular) epithelium-free areas. (B) Rat thymic cortex was stained with anti-CD205 antibody or anti-CD103 antibody followed by Alexa594-conjugated anti-mouse IgG, Alexa488-conjugated ED18 and anti-rat MHC II conjugated with Alexa647. Arrowheads indicate ED18^−^CD205^−^MHC II^+^ or ED18^−^CD103^+^MHC II^+^ cells.
(TIF)

Figure S2 **Characterization of mouse medullary thymic epithelial cells 1: relationship between ED18 and ED21 staining.** A section of a thymus from a C57BL/6 mouse was stained and pictures are displayed in the same manner as in Figure 3, except for Alexa647-conjugated anti-mouse MHC II antibody.
(TIF)

Figure S3 **Characterization of mouse medullary thymic epithelial cells 2: relationship between keratin expression and ED18/ED21.** Sections of a thymus from a C57BL/6 mouse were stained and pictures are displayed in the same manner as in Figure 4, except for Alexa647-conjugated anti-mouse MHC II antibody.
(TIF)

Figure S4 **Characterization of mouse medullary thymic epithelial cells 3: UEA-1 binding to mTEC subpopulations.** A section of a thymus from a C57BL/6 mouse was stained and pictures are displayed in the same manner as in Figure 5A, except for Alexa647-conjugated anti-mouse MHC II antibody.
(TIF)

Figure S5 **Expression of functional molecules in mouse mTEC1 and mTEC2 subsets.** Sections of a thymus from a C57BL/6 mouse were stained and pictures are displayed in the same manner as in Figure 6A.
(TIF)

Figure S6 **Epitope Analysis of ED monoclonal antibodies.** Proteins in whole rat thymic lysate were subjected to western blot analysis. Unconjugated ED18, ED19, and ED21 followed by peroxidase-conjugated anti-mouse IgM were used.
(TIF)
