# Supplementary figures and images for: Three Distinct Subsets of Thymic Epithelial Cells in Rats and Mice Defined by Novel Antibodies
Source: PLoS One. 2014 Oct 21;9(10):e109995. doi: 10.1371/journal.pone.0109995 (PMC4204869; doi:10.1371/journal.pone.0109995)

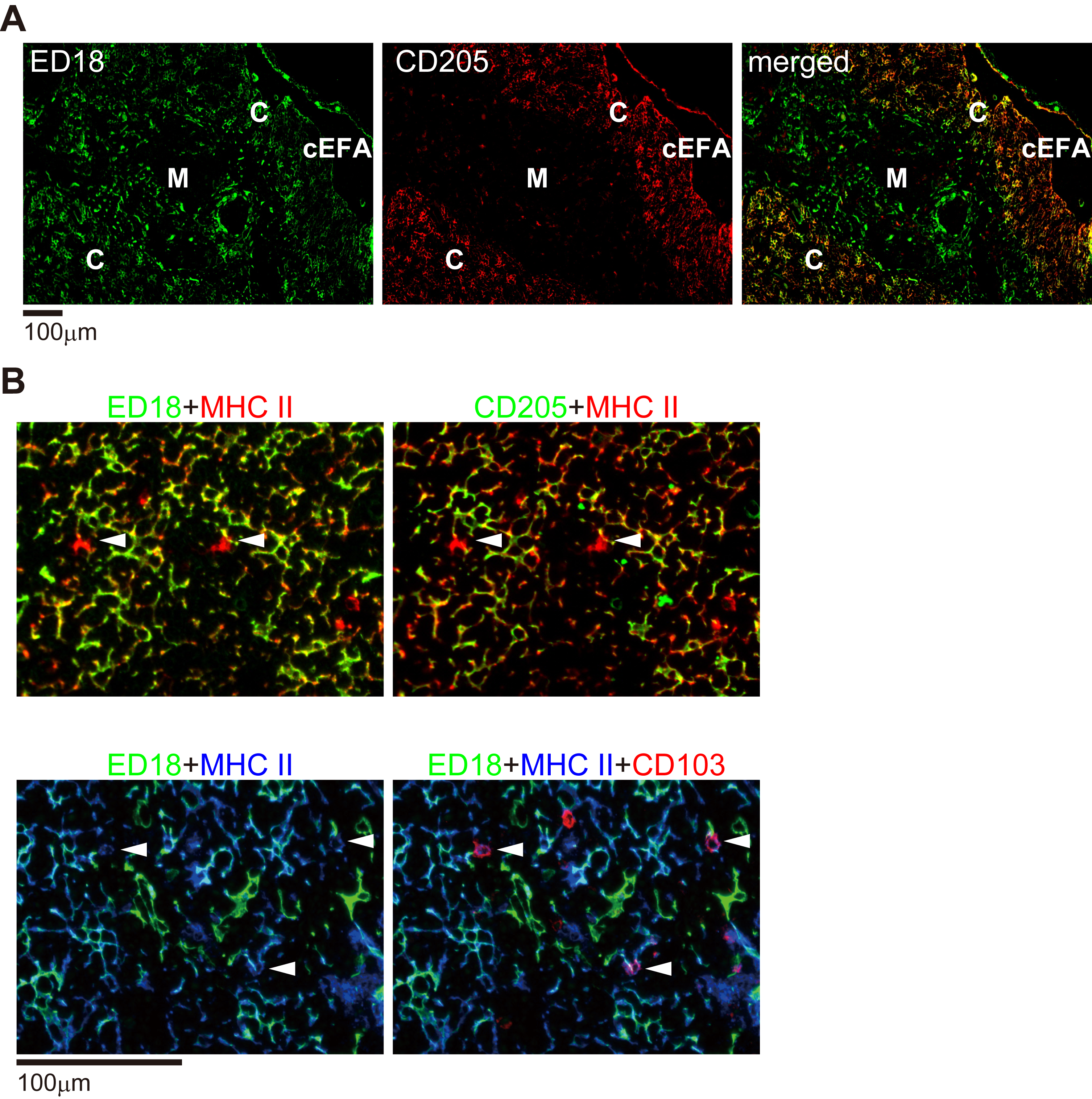

Supplement: Figure S1 — CD205 expressed on cortical thymic epithelial cells in the cortex and dendritic cells in the medulla. (A) A rat thymus was stained with anti-CD205 followed by Alexa594-conjugated anti-mouse IgG and Alexa488-conjugated ED18. C, cortex; M, medulla; cEFA, cortical (subcapsular) epithelium-free areas. (B) Rat thymic cortex was stained with anti-CD205 antibody or anti-CD103 antibody followed by Alexa594-conjugated anti-mouse IgG, Alexa488-conjugated ED18 and anti-rat MHC II conjugated with Alexa647. Arrowheads indicate ED18−CD205−MHC II+ or ED18−CD103+MHC II+ cells. (TIF) [file pone.0109995.s001.tif]

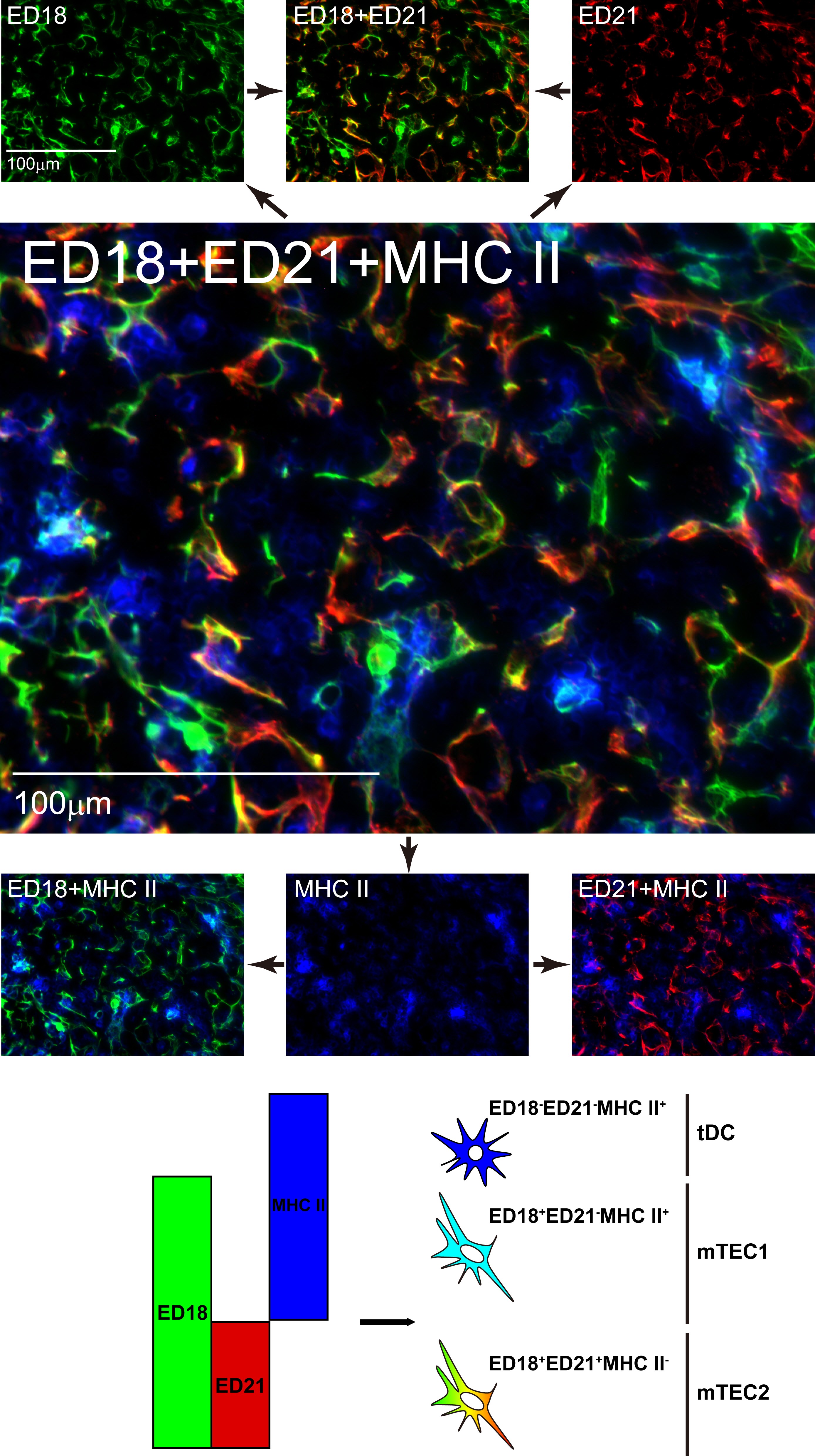

Supplement: Figure S2 — Characterization of mouse medullary thymic epithelial cells 1: relationship between ED18 and ED21 staining. A section of a thymus from a C57BL/6 mouse was stained and pictures are displayed in the same manner as in Figure 3, except for Alexa647-conjugated anti-mouse MHC II antibody. (TIF) [file pone.0109995.s002.tif]

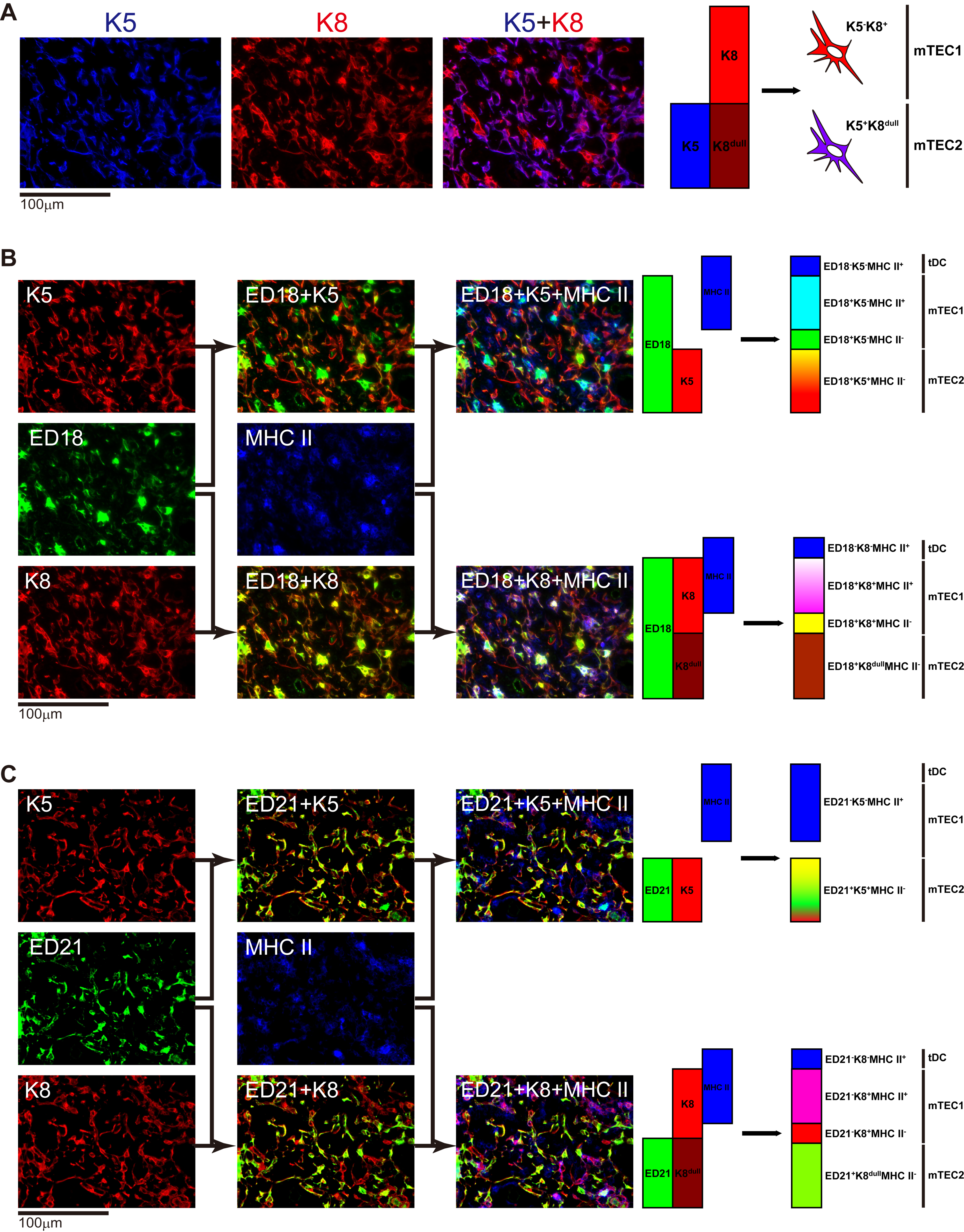

Supplement: Figure S3 — Characterization of mouse medullary thymic epithelial cells 2: relationship between keratin expression and ED18/ED21. Sections of a thymus from a C57BL/6 mouse were stained and pictures are displayed in the same manner as in Figure 4, except for Alexa647-conjugated anti-mouse MHC II antibody. (TIF) [file pone.0109995.s003.tif]

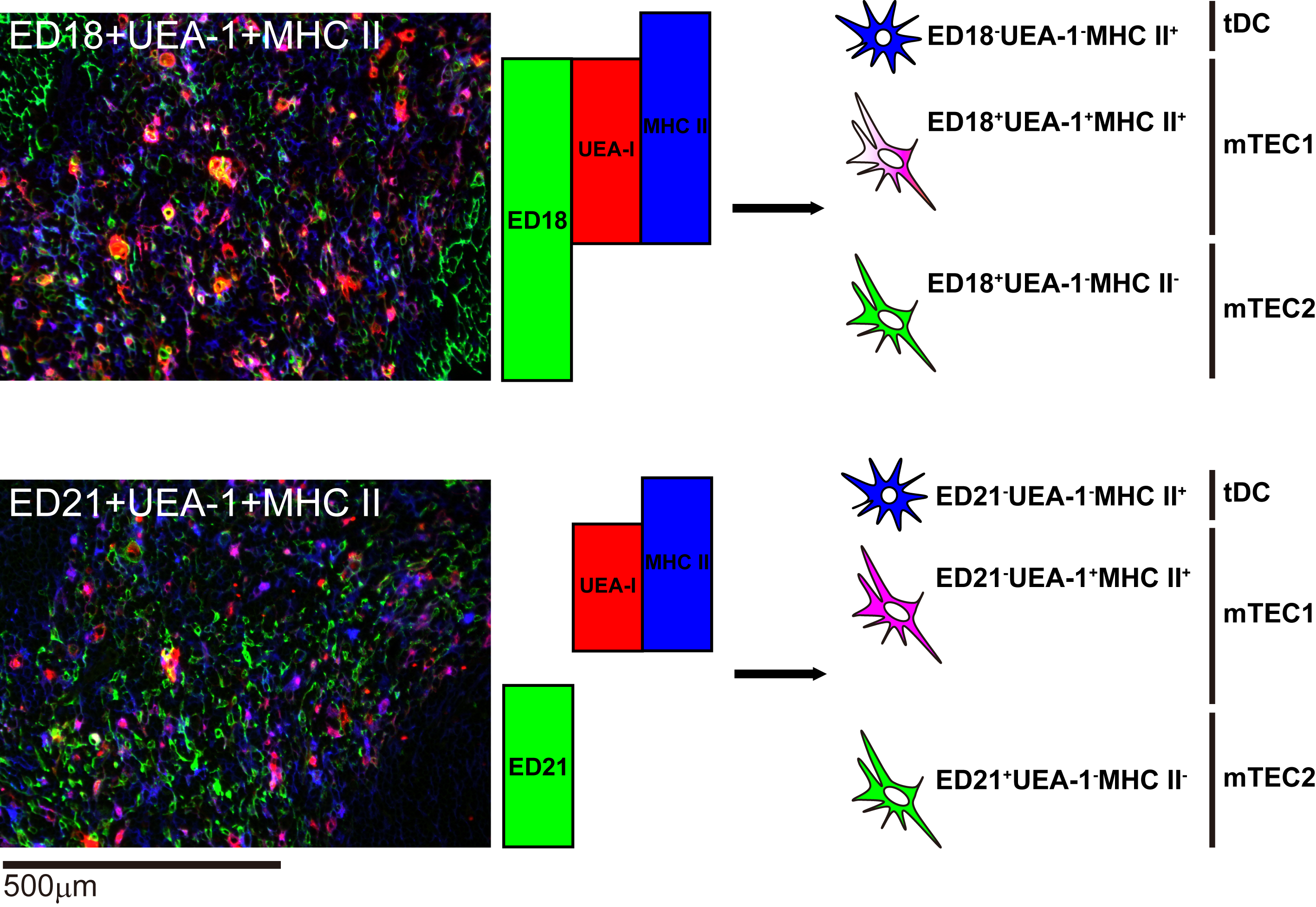

Supplement: Figure S4 — Characterization of mouse medullary thymic epithelial cells 3: UEA-1 binding to mTEC subpopulations. A section of a thymus from a C57BL/6 mouse was stained and pictures are displayed in the same manner as in Figure 5A, except for Alexa647-conjugated anti-mouse MHC II antibody. (TIF) [file pone.0109995.s004.tif]

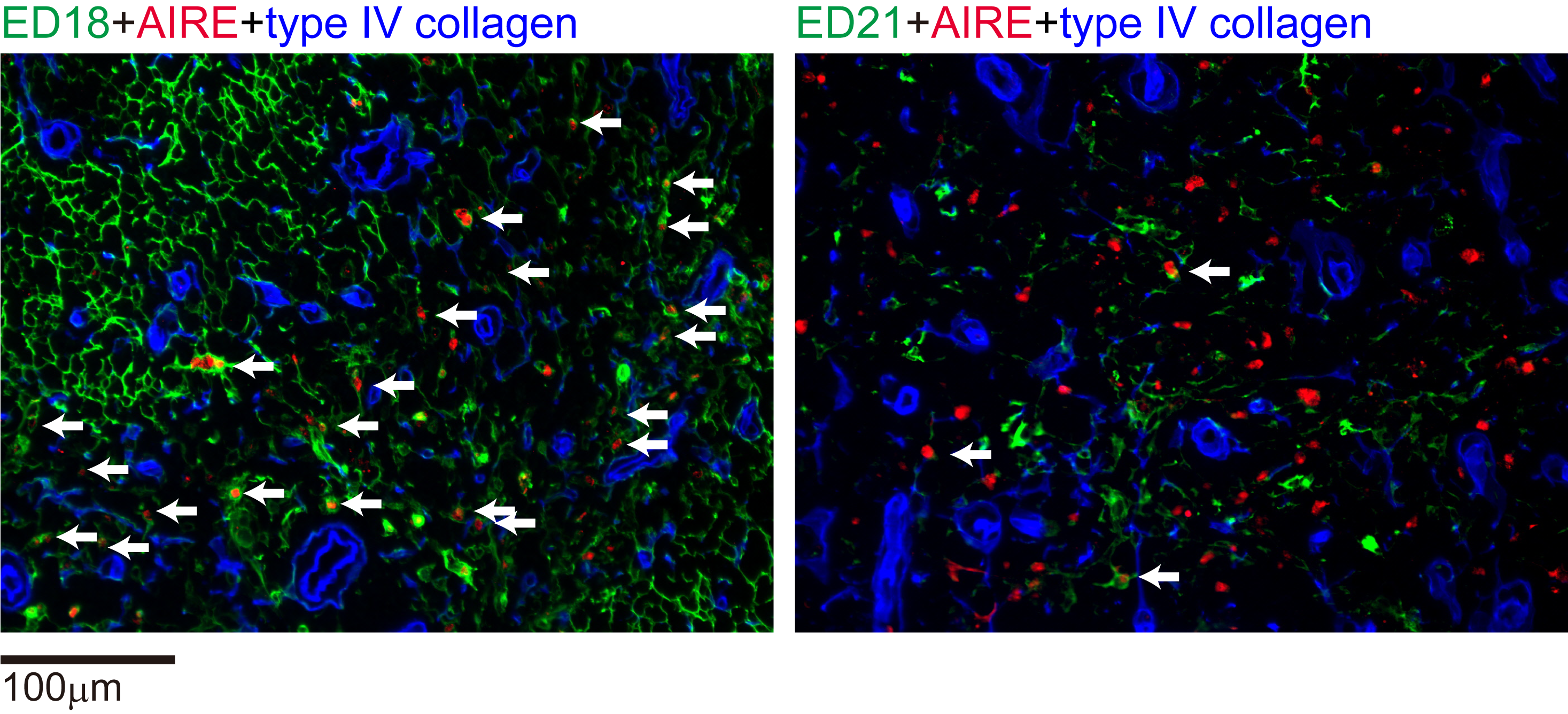

Supplement: Figure S5 — Expression of functional molecules in mouse mTEC1 and mTEC2 subsets. Sections of a thymus from a C57BL/6 mouse were stained and pictures are displayed in the same manner as in Figure 6A. (TIF) [file pone.0109995.s005.tif]

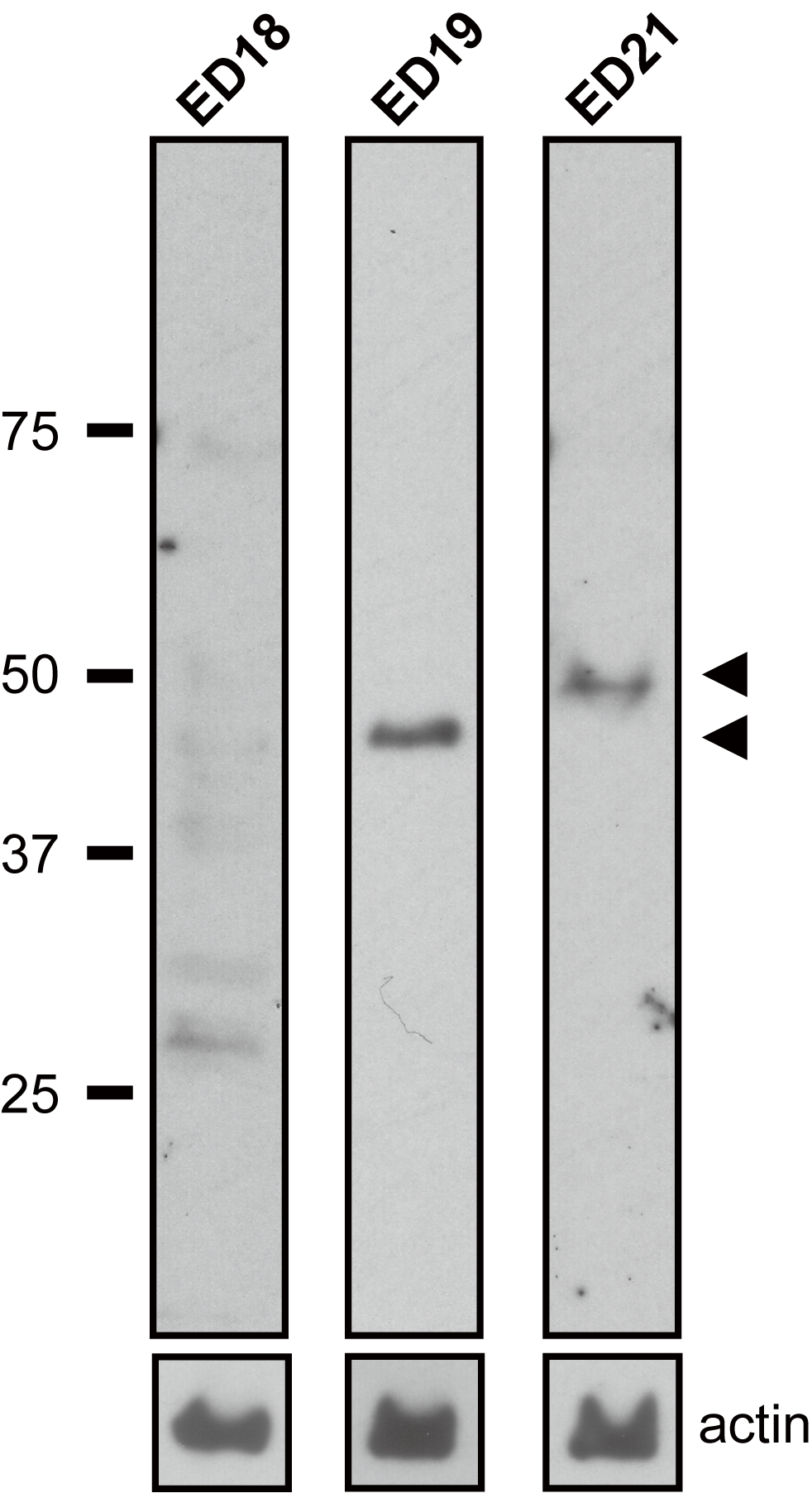

Supplement: Figure S6 — Epitope Analysis of ED monoclonal antibodies. Proteins in whole rat thymic lysate were subjected to western blot analysis. Unconjugated ED18, ED19, and ED21 followed by peroxidase-conjugated anti-mouse IgM were used. (TIF) [file pone.0109995.s006.tif]
